# Renal infarction due to polyarteritis nodosa in a patient with angioimmunoblastic T-cell lymphoma: a case report and a brief review of the literature

**DOI:** 10.1186/1746-1596-7-50

**Published:** 2012-05-08

**Authors:** Maria Raffaella Ambrosio, Bruno Jim Rocca, Alessandro Ginori, Monica Onorati, Alberto Fabbri, Mario Carmellini, Stefano Lazzi, Sergio Tripodi

**Affiliations:** 1 Department of Human Pathology and Oncology, Pathological Anatomy Section, University of Siena, via delle Scotte, Siena, 6 - 53100, Italy; 2 Unit of Hematology and Transplant, University of Siena, Siena, Italy; 3 Unità Operativa Chirurgia dei Trapianti, Azienda Ospedaliera Universitaria Senese, Senese, Italy

**Keywords:** Renal infarction, Polyarteritis nodosa, T-cell lymphoma

## Abstract

Angioimmunoblastic T-cell lymphoma is one of the most common subtypes of peripheral T-cell lymphoma (15-20% of all cases), accounting for approximately 1-2% of all non-Hodgkin lymphomas. It often presents autoimmune phenomena including hemolytic anemia, thrombocytopenia, glomerulonephrities and circulating immune complexes. Polyarteritis nodosa is an autoimmune disease characterized by necrotizing vasculitis of medium vessels, which rarely develops in association with hematological malignant disorders. Herein we report the case of a 40-year-old man who underwent lymph node biopsy in the suspicious of sarcoidosis. On the basis of histological and immunohistochemical findings, the diagnosis of angioimmunoblastic T-cell lymphoma was performed. The patient was successfully treated with cytarabine-based regimen for 6 cycles. Three months after the initial diagnosis of angioimmunoblastic T-cell lymphoma, a whole body computed tomography showed a lesion in the lower pole of the left kidney. Renal cell carcinoma was suspected, thus a nephrectomy was carried out. The histological findings were compatible with polyarteritis nodosa. To the best of our knowledge, the association between polyarteritis nodosa and angioimmunoblastic T-cell lymphoma has been described only once. This relation may be secondary to the induction of an autoimmune phenomenon by the lymphoma with the formation of circulating immune complexes, leading to vessels walls injury. A careful evaluation is needed in the management of angioimmunoblastic T-cell lymphoma patients with signs of renal failure in order to avoid delay of treatment and organ damage.

## Background

The World Health Organization (WHO) classification of tumors of hematopoietic and lymphoid tissues defines the Angioimmunoblastic T-cell lymphoma (AITL) as one of the more common specific subtypes of peripheral T-cell neoplasms, accounting for approximately 15-20% of all cases, or 1-2% of all non-Hodgkin lymphomas [1]. It occurs in middle age and elderly, with an equal incidence in males and females and has an aggressive clinical behavior, often with fever, skin rash, generalized lymphadenopathy, hepatosplenomegaly and autoimmune phenomena including hemolytic anemia, thrombocytopenia, glomerulonephrities and circulating immune complexes (CIC). Histologically, it is characterized by polymorphous infiltrate involving lymph nodes, and prominent proliferation of high endothelial venules and follicular dendritic cells. During the clinical course of a lymphoma, many lesions (not only direct invasion by neoplastic cells) may affect the kidney as broad phenomenon disorders [2]. This finding is more common in Hodgkin’s disease rather than non-Hodgkin’s lymphoma. During the clinical course of AITL, proliferative glomerulonephritis [3], minimal-change disease [4,5], A-type amyloidosis [2], acute renal failure [6], immunoglobulin (Ig)M-λ glomerular thrombi, and membranoproliferative glomerulonephritis-like lesions [7], myeloma-like kidney [8], direct invasion by lymphoma cells [9], interstitial nephritis [10,11], vasculitis [12,13], nephrocalcinosis [14], Fanconi syndrome [15] and nephrotic syndrome due to membranous nephropathy (MN) [16] may be rarely observed.To the best of our knowledge, there is only one previous case of AITL associated with polyarteritis nodosa (PAN) [17]. Herein, we report a very unusual complication in AITL, a renal necrotic lesion due to vasculitis with the appearance of PAN, clinically misdiagnosed as renal carcinoma, highlighting the possible pathogenic mechanism.

## Case presentation

### Clinical summary

**Figure 1:**
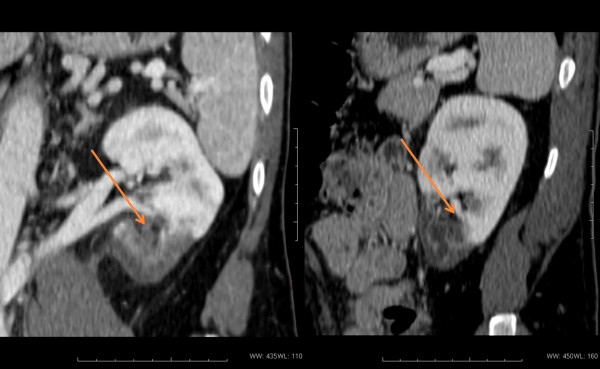
**TC scan findings.** A lesion of 45 mm in the upper pole of the left kidney is shown.

A 40 year-old-male patient presented to his general practitioner with a 3-weeks history of myalgia, arthralgia, fever (37.5°C) and cough which persisted despite a broad-spectrum antibiotic therapy. For this, he underwent a chest X-ray examination which showed bilateral lung nodules and enlarged mediastinal lymph nodes, with a clinical picture consistent with sarcoidosis. The patient was admitted to the Pneumologic Unit of Siena University Hospital. On admission, physical examination showed generalized lymphadenopathy, splenomegaly and skin rash. The laboratory data are summarized in Table 1. The blood count, liver and renal function, as well as angiotensin converting enzyme (ACE), were within the reference range. Eosinophilia and hypergammaglobulinemia were not found whereas C-reactive protein, lactate dehydrogenase, β2-microglobulin and serum immunoglobulins were increased. Since the clinical picture was doubtful, the patient underwent biopsy of the axillary lymph node, the maximum diameter of which was 3 cm. On the basis of clinical presentation and histological findings, the diagnosis of angioimmunoblastic T-cell lymphoma stage IVB was made according to the criteria of WHO classification [1]. The patient received a combination chemotherapy with pegylated liposomal doxorubicin (30 mg/m^2^, day 1), cytarabine (2 g/m^2^ day 2–3) and dexamethasone (40 mg day 1–4) every 21 days. After two cycles, the patient presented a marked improvement of the systemic symptoms and no superficial lymphadenopathies were observed. After 3 months a re-stage whole body computed tomography (CT)-scan was performed which showed the disappearance of all previous pathologic findings, but evidenced a lesion of 45 mm in the lower pole of the left kidney (Figure 1). Such finding was later confirmed by an ultrasound scan, that showed, at the color Doppler exam, a vascular pattern consistent with renal cell carcinoma. The patient was readmitted to the Hospital in order to perform a nephrectomy. The final diagnosis was renal infarction due to PAN, according to Carlson [18]. The patient had negative rheumatoid factor, HLA-B27, streptolysin O, anti-nuclear, anti-cardiolipin, anti-DNA, anti-smith, anti-RNP, anti-SSA, anti-SSB, anti-neutrophil cytoplasmic and anti-lupus anticoagulant antibodies. Serologies and PCR for both HBV and HCV were negative. The histological diagnosis of PAN was also confirmed by clinicians according to American College of Rheumatology (ACR) criteria [19]. After six days, the patient was discharged from hospital without complications. He completed the chemotherapy induction program (4 cycles) and underwent consolidation with high-dose chemotherapy and autologous stem cells transplantation. He is well five years after surgery. 

**Table 1:** Summary of laboratory data

	**Our values**	**Normal range**
Hb	12,2 g/dl	14-18 g/dl
RBC	5,3 × 10^6^/mm^3^	4,5-6 × 10^6^/mm^3^
WBC	6,6 × 10^3^/mm^3^	4-8 × 10^3^/mm^3^
Hematocrit	45%	40-52%
MCV	90 μm^3^	83-93 μm^3^
MCHC	34 g/dl	32-36 g/dl
Platelet count	225 × 10^3^/mm^3^	150-350 × 10^3^/mm^3^
AST	10 UI/l	0-35 UI/l
ALT	10 UI/l	0-35 UI/l
LDH	552 U/l	120-240 U/l
Total protein	8,6 g/dl	6,5-8,0 g/dl
Albumin	42,3 g/l	35,2-50,4 g/l
β_2_-microglobulin	5.2 mg/dL	0,1-0,2 mg/dL
Blood urea nitrogen	32 mg/dl	10-50 mg/dl
Creatinine	0,9 mg/dl	0-1,3 mg/dl
Blood glucose	96 mg/dl	70-110 mg/dl
C-reactive protein	35 mg/l	0-5 mg/l
ACE	15 U/l	< 40 U/l
IgA	1150 mg/dl	90-450 mg/dl
IgG	2245 mg/dl	80-1800 mg/dl
IgM	135 mg/dl	60-250 mg/dl

*Hb* Haemoglobin, *RBC* red blood cells count, *WBC* white blood cells count, *MCV* mean corpuscular volume, *MCHC* mean cell haemoglobin concentration, *AST* serum aspartate aminotransferase, *ALT* alanine aminotransferase, *LDH* lactate dehydrogenase, *ACE* angiotensin converting enzyme.

### Pathologic findings

**Figure 2:**
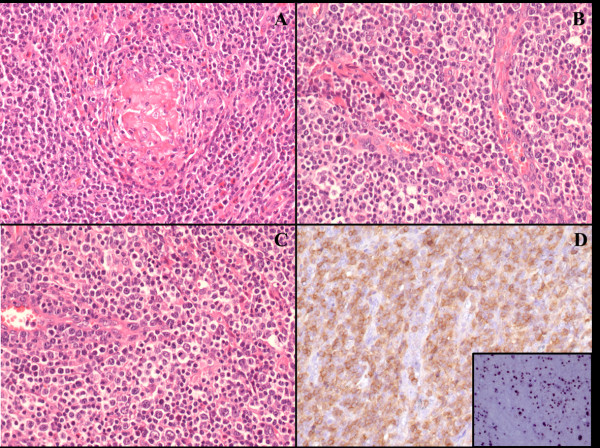
**Axillary lymph node morphology.** Effacement of lymph node architecture with burnt-out follicles (**A**) and marked vascular proliferation (**B**) was observed. The neoplastic cells show clear-to-pale cytoplasm, distinct cell membrane and minimal atypia (**C**); they mainly express CD4 (**D**). EBV-positive B cells are present (inset, **D**). [**A**-**C:** Haematoxylin–Eosin (H&E); Original Magnification (O.M.): 40x; **D:** CD4 stain, O.M.: 40x; **D**, inset: EBER in situ hybridization, O.M.: 40x.

**Figure 3:**
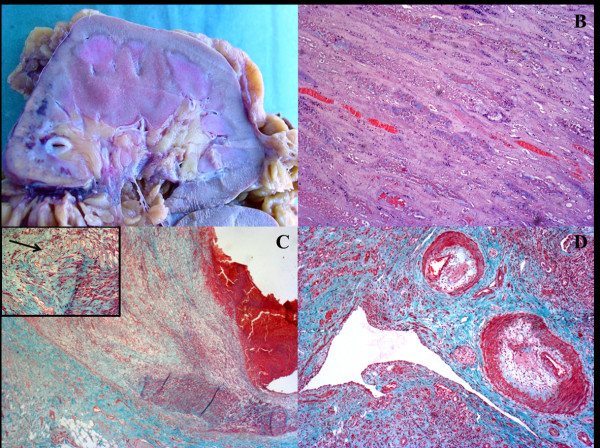
**Renal infarction.** Gross morphology shows a large pale lesion of the lower pole (**A**). Histological examination shows coagulative necrosis of renal parenchyma (**B**), aneurysmal distension of the arterial wall (**C**) and rupture of the internal elastic lamina (**C**, inset, arrow). Some vascular lumina are obliterated by fibrous stroma and recanalized by thin vascular channels (**D**). (**B**-**D:** Masson stain; O.M.: 40x; 20x; 40x).

Serial sections of both axillary lymph node and left kidney were performed, routinely processed, stained with haematoxylin and eosin and examined by light microscopy. Histologically, the lymph node architecture was partially effaced by polymorphic cellular infiltration, burnt-out follicles (Figure 2A) and proliferation of numerous arborizing high-endothelial venules (Figure 2B). An expansion of paracortex was observed, which was diffusely infiltrated by a polymorphous population of small to medium-sized lymphocytes, with distinct cell membranes, clear to pale cytoplasm, and mild irregular nuclei (Figure 2C). The neoplastic population was admixed with small reactive lymphocytes, eosinophils, plasma cells, histiocytes and numerous follicular dendritic cells. Few large immunoblast-like lymphoid cells with large distinct nuclei and clear cytoplasm were observed intermingled with lymphocytes. In addition, scattered Reed-Sternberg (RS)-like cells with irregular multilobated nuclei and large eosinophilic nucleoli were present in the node. Immunohistochemically, the neoplastic T-cells were positive for CD45Ro, CD3, CD10, LANA-1 and LMP and expressed mostly the CD4 antigen (Figure 2D), although numerous reactive CD8 positive T-cells were present. CD20, CD79a, PAX-5, CD56, MUM-1 and CD30 were negative. The large immunoblast-like cells and the scattered RS-like cells showed immunoreactivity to CD20, CD79a and CD30. The proliferation of follicular dendritic cells highlighted by CD21 and CD23 was prominent throughout the node, and entrapped high-endothelial venules. By means of in situ hybridization RNAs (EBERs), EBER-positive signals were observed in scattered large B immunoblasts and RS-like cells (Figure 2D, inset). Molecular studies showed monoclonal rearrangement of T-cell receptor genes and polyclonal rearrangement of immunoglobulin heavy chain (IgH) receptor. Macroscopic examination of the left kidney specimen showed a large pale area at the lower pole, approximately 4 cm in maximum diameter with a triangular morphology, centered on the renal cortex and consistent with an infarcted area (Figure 3A). Coagulative necrosis of renal parenchyma (Figure 3B) and multiple segmentary inflammatory lesions of small and middle renal arteries were observed on histological examination. Masson and Giemsa stains showed rupture of internal elastic lamina with aneurysmal collapse of the arterial wall (Figure 3C). Some vascular lumina were obliterated by fibrous stroma and sometimes recanalized by thin vascular channels (Figure 3D). There was no infiltration by neoplastic T-cells.

## Discussion

AITL is a disease of the middle-aged and elderly people but it rarely occurs in the fourth decade, as it has happened in our patient [20,21]. No standard of care has been established and the prognosis is poor, with a median survival of less than three years [1]. Responder patients seem to benefit from high dose chemotherapy and autologous stem cell transplantation. AITL was previously considered an atypical reactive process, named angioimmunoblastic lymphadenopathy [22]; currently, overwhelming evidence deriving from molecular and cytogenetic studies suggests that AITL rises *de novo* as a peripheral T-cell lymphoma [1]. The nearly constant association with EBV has suggested a possible role for the virus in the etiology, possibly through an antigen-driven process. However, the neoplastic T cells are EBV negative. They express most pan T-cell antigens such as CD3, CD2 and CD5 and, in vast majority of the cases, CD4, although numerous reactive CD8 positive T-cells are often present. In 60-100% of the cases, the tumour cells express CD10, CXCL13 and PD-1. In our case, not only histological examination of lymph node demonstrated morphological features of AITL, but also the presence of T-cell lineage with aberrant CD4 and CD10 expression strongly supported the diagnosis [22]. Moreover, TCR genes showed clonal rearrangement. Immunoblast-like cells and RS-like cells showed polyclonal IgH rearrangement, thus a concomitant large B cell lymphoma (a well-known complication in AITL) was excluded.

A review of the literature has demonstrated that a wide spectrum of renal lesions (not only direct infiltration by neoplastic cells) in glomeruli, tubule-interstitium and vessels can develop in patients with AITL and angioimmunoblastic lymphadenopathy [23-26]. Clinico-pathological features of the patients are listed in Table 2. To the best of our knowledge, only one case of AITL associated with PAN [17], has been previously described. It concerned an elderly (71 years) man, who developed an intraperitoneal hemorrhage two months after the diagnosis of AITL. PAN is an autoimmune necrotizing systemic vasculitis that preferentially involves small and medium-sized arteries, with signs and symptoms resulting from infarction and scarring of the affected organ. It often starts with non specific symptoms and laboratory features [27]. Any age group may be affected, but it is commonly seen in people between the ages of 40 and 60, as in our case. The most frequent visceral manifestations involve kidney (93.4%), heart (72%), and gastrointestinal tract (57.4%) [28]; moreover, several reports indicate that the disease may affect also testis and prostate [29]. Currently, the most widely used vasculitis classification system is that of the ACR which is based predominantly on clinical findings. Histological diagnosis of vasculitis is performed according to the Chapel Hill Consensus Conference criteria [30] and to the more recent scheme suggested by Carlson [18]. The possible mechanism that may explain the relation between AITL and PAN is represented by the induction of an autoimmune phenomenon by the lymphoma. It is well known that AITL cells produce cytokines such as interleukin 6 [31] and TNF-α [16], stimulating polyclonal B-cells and plasma cells to secrete antibodies forming circulating immune complexes (CIC). CIC complete the complement cascade, releasing vasoactive substances and chemotactic factors that cause accumulations of inflammatory cells and release of lysosomal enzymes by neutrophils, leading to injury of vessels walls and thrombosis [17]. In the patient here presented, only serum IgG value and C-reactive protein were elevated whereas platelet count and CIC were normal. Throughout the course of the disease, platelet count decrease and CIC increment was documented. The main diagnostic difficulty was represented by the absence of signs and symptoms of vasculitis; moreover, echo-color-doppler and CT scan findings led to a misdiagnosis of renal carcinoma. Only histological examination of the kidney which showed necrosis of parenchyma and multiple segmentary vasculitis involving small and middle renal arteries allowed the correct diagnosis of PAN which was later confirmed by clinicians. 

**Table 2:** Clinicopathological features of patients with AITL developing renal involvement

**Author**	**Sex**	**Age**	**Type of renal lesion**	**Interval (months)**	**Treatment**	**Clinical outcome**
Wood and Harkins [13]	M	76	Diffuse proliferative glomerulonephritis	0	Corticosteroid, cyclophosphamide	Dead for lymphoma
Wood and Harkins [13]	M	79	Minimal change disease	0	Dialysis	Dead for renal failure
Bhat et al [8]	F	77	Acute renal failure with Bence-Jones proteinuria	4	None	Dead for sepsis
Platzer et al [11]	M	64	Renal failure	0	Prednisolone	CR
Bello et al [15]	M	61	Fanconi syndrome	0	Hydrocortisone	CR
Bignon et al [23]	M	70	Dysproteinaemia	0	n.a.	n.a.
Yamazaki et al [26]	M	72	Endocapillary proliferative glomerulonephritis	0	Vincristine, prednisolone	Dead for alimentary tract bleeding
Nakamoto et al [10]	M	40	Acute interstitial nephritis	16	Prednisolone, cyclophosphamide	At 60-month follow-up, no signs of relapse
Duwaji et al [28]	M	71	Proliferative glomerulonephritis	2	CHOP regimen	Dead for sepsis
Lim et al [33]	M	33	Amyloidosis	12	CHOP regimen	At 12-month follow-up, no signs of relapse
Hamidou et al [12]	M	56	Vasculitis	0	CHOP regimen	Dead for renal failure
De Samblanx et al [2]	M	67	Proliferative glomerulonephritis	0	CHOP regimen	At 12-month follow-up, no signs of relapse
Goto et al [9]	M	73	Direct invasion by lymphoma	0	CHOP regimen	At 20-month follow-up, no signs of relapse
Miura et al [7]	M	70	IgM-λ glomerular thrombi	2	CHOP regimen	At 3-month follow-up, no signs of relapse
Tagashi et al [16]	M	21	Nephrotic syndrome	0	CHOP regimen	At 36-month follow-up, no signs of relapse

*CR* complete remission; *n.a.* not available, *CHOP* Cyclophosphamide, doxorubicin, vincristine, prednisolone.

## Conclusion

A careful evaluation is needed in the management of AITL patients which may exhibit immunodeficiency and autoimmunity secondary to the neoplastic process [32]. Herein we report the first case of PAN presenting at onset with renal involvement, sequential to AITL, in a 40 year-old man. PAN has rarely been reported in association with AITL but, although infrequent, clinicians should keep in mind the possibility of an autoimmune disorder involving kidney, in case signs of renal failure develop. Renal biopsy and angiography are necessary in order to avoid delay of treatment and organ damage.

## Consent

Written informed consent was obtained from the patient for publication of this Case Report and any all accompanying images. A copy of the written consent is available for review by the Editor-in-Chief of this journal.

## Abbreviations

AITL, Angioimmunoblastic T-cell lymphoma; CT, Computed tomography; PAN, Polyarteritis nodosa; WHO, World Health Organization; CIC, Circulating immune complexes; Ig, Immunoglobulins; ACE, Angiotensin converting enzyme; ACR, American College of Rheumatology; RS, Reed-Sternberg; EBER, EBV-encoded RNA; EBV, Epstein-Barr virus; AILD, Angioimmunoblastic lymphadenopathy with dysproteinemia.

## Competing interest

The Authors declare that they have no competing interests.

## Authors’ contributions

MRA wrote the paper; BJR performed analysis of the histological sections; AG and MO carried out the immunoassays; AF and MC made contributions to acquisition of clinical data; SL contributed his expertise in the field and fruitful discussion; SAT coordinated the work and gave final approval of the version to be published. All authors read and approved the final manuscript.
